# Plant extracts from Cameroonian medicinal plants strongly inhibit hepatitis C virus infection *in vitro*

**DOI:** 10.3389/fmicb.2015.00488

**Published:** 2015-05-15

**Authors:** Borris R. T. Galani, Marie-Emmanuelle Sahuc, Frederic N. Njayou, Gaspard Deloison, Pierre Mkounga, William F. Feudjou, Priscille Brodin, Yves Rouillé, Augustin E. Nkengfack, Paul Fewou Moundipa, Karin Séron

**Affiliations:** ^1^Laboratory of Pharmacology and Toxicology, Department of Biochemistry, Faculty of Science, University of Yaoundé IYaoundé, Cameroon; ^2^Department of Biological Sciences, Faculty of Science, University of NgaoundéréNgaoundéré, Cameroon; ^3^Molecular and Cellular Virology, Center for Infection and Immunity of Lille, Inserm U1019 – CNRS UMR 8204, Institut de Biologie de Lille, Pasteur Institute of Lille, University of LilleLille, France; ^4^Laboratory of Physical Chemistry and Phytochemistry, Department of Organic Chemistry, Faculty of Science, University of Yaoundé IYaoundé, Cameroon

**Keywords:** hepatitis C, plant extract, entry inhibitor, traditional medicine, natural compounds

## Abstract

According to some recent studies, Cameroon is one of the sub-Saharan African countries most affected by hepatitis C, with low access to the standard therapy based on the combination of pegylated interferon and ribavirin. A first ethnobotanical survey, conducted in the Western region of Cameroon, reported the use of several medicinal plants in traditional medicine for the healing of liver-related disorders. Crude organic extracts of five plants surveyed were prepared and their effect against hepatitis C virus (HCV) infection investigated. The HCV JFH1 strain cell culture system HCVcc was used. The antiviral activity was quantified by immunofluorescent labeling of HCV E1 envelope protein at 30 h post-infection in the presence of the plant extracts. Active compounds were then tested in time course infection experiments. Dose-response and cellular toxicity assays were also determined. Three extracts, methanol extracts from roots of *Trichilia dregeana*, stems of *Detarium microcarpum* and leaves of *Phragmanthera capitata,* showed anti-HCV activity, with half-maximal inhibitory concentration of 16.16, 1.42, and 13.17 μg/mL, respectively. Huh-7 cells were incubated with the extracts for 72 h and it appears that *T. dregeana* extract is not toxic up to 200 μg/mL, *D. microcarpum* up to 100 μg/mL and *P. capitata* up to 800 μg/mL. All the three extracts showed a strong inhibition of HCV entry and no effect on replication or secretion. Taken together, these results showed that extracts from Cameroonian medicinal plants are promising sources of anti-HCV agents.

## Introduction

Hepatitis C virus (HCV) infection is one of the major causes of chronic hepatitis, and related complications such as steatosis, fibrosis, cirrhosis, and hepatocellular malignancies (Sangiovanni et al., 2006). According to WHO, approximately 130–170 millions people are chronically infected by HCV globally and over a million new cases occur each year. In Africa, a recent study reported that more than 28 millions people are chronically infected by the virus (Lavanchy, 2011). However, the prevalence data are still incomplete and vary considerably from one population studied to another. In Cameroon, the prevalence of hepatitis C is high and was estimated to about 13% of the whole population (Nerrienet et al., 2005), with a great predominance of genotypes 1, 2, and 4 (Ndjomou et al., 2002). Phylogenetic analysis performed with viral isolates coming from Cameroonian patients infected by genotype 1 strains, revealed a high diversity in this genotype. Seven genotype 1 subtypes (1a–1c, 1e, 1g, 1h, 1l) and two unclassified lineages were recently identified and characterized (Li et al., 2013).

To date, there is no vaccine against hepatitis C and the great diversity of viral isolates makes its development difficult. Over the last decade, the standard therapy against hepatitis C was based on the combination of pegylated-interferon α-2a or 2b (Peg-IFNα) with ribavirin (RBV), a guanosine analog. This treatment allowed reaching a sustained virological response (SVR) of 76–84% in genotype 2-infected patients and only 40–50% in genotype 1-infected patients (Mauss et al., 2013). Nevertheless, a poor therapeutic response (56%) was observed with this combination in genotype 2-HCV infected Cameroonians (Njoya et al., 2013). In 2011, two NS3/4A protease inhibitors (Boceprevir and Telaprevir) were approved by the American Agency for Food and Drug Administration (FDA) and are now used in association with the standard bitherapy to improve the viral clearance rates in genotype 1 infected patients. Cure rates of approximately 70% were achieved with this triple combination in these patients. Recently, in 2013, two more effective compounds were approved to reinforce the therapeutic arsenal: Simeprevir, a protease inhibitor and Sofosbuvir a polymerase inhibitor. In October 2014, FDA approved a new drug for hepatitis C treatment: Harvoni which is a combination of Sofosbuvir and Ledipasvir (Keating, 2015). This drug is used for a 12-week course treatment and has the advantage that no IFN or RBV is needed and higher SVR rates are achieved. However, if these molecules are effective, they are not really accessible for most-infected patients especially in low-income countries, and less than 10% of identified patients are cured with the standard bitherapy. Moreover the side effects of Peg-IFNα and these inhibitors as well as emergence of resistant HCV variants remain a problem. It is currently thought that combinations of multiple direct antiviral agents (DAAs) without Peg-IFNα may lead to higher SVR rates. Efforts are then needed to identify such ingredients in medicinal plants.

A first ethnobotanical survey, conducted in the Western region of Cameroon, reported the use of 53 medicinal plants in the Bamun folk medicine for the management of jaundice and other hepatic disorders. Antioxidant properties of these plants were demonstrated *in vitro* on microsomal lipid peroxidation and protein oxidation (Njayou et al., 2008). Recently, we found that the crude extract and fractions of *Entada africana*, one of these plants, possessed an anti-HCV activity in genotype 1b replicons systems (Galani Tietcheu et al., 2014). However with most of these plants, no effect on HCV infection has yet been reported. Recent studies highlighted the efficacy of molecules from plant origin as anti-HCV agents (Calland et al., 2012b). Among them, epigallocatechin gallate (EGCG; Ciesek et al., 2011; Calland et al., 2012a; Chen et al., 2012), ladanein (Haid et al., 2012) and cucurmin (Anggakusuma et al., 2013) are the most promising inhibitors. Due to their low cost of production, extracts, or molecules isolated from plants used in traditional medicine might be useful in the therapeutic arsenal against hepatitis C in low income countries. In this study, we tested seven different extracts from five plants and report the anti-HCV activity of three Cameroonian plant extracts by the use of HCV cell culture system (HCVcc), representing the complete infectious cycle of the virus.

## Material and Methods

### Chemicals

Dulbecco’s modified Eagle’s medium (DMEM), glutamax-I, goat, and fetal calf sera were purchased from Invitrogen (Carlsbad, CA, USA). 4′,6-Diamidino-2-phenylindole (DAPI) was from Molecular Probes (Life technologies). EGCG was from Calbiochem (Merck Chemicals, Darmstadt, Germany). Stocks were resuspended in dimethyl sulfoxide (DMSO) at 0.5 M for EGCG and 250 mg/mL for plant extracts. Boceprevir was kindly provided by Philippe Halfon (Hôpital Européen, Laboratoire Alphabio, Marseille, France).

### Antibodies, Cells, and Culture Conditions

Mouse anti-E1 monoclonal antibody (MAb) A4 (Dubuisson et al., 1994) was produced *in vitro*. Cy3-conjugated goat anti-mouse IgG was from Jackson Immunoresearch (West Grove, PA, USA). Huh-7 hepatoma cells (Nakabayashi et al., 1982) were grown in DMEM supplemented with glutamax-I and 10% fetal calf serum, in an incubator at 37°C with 5% CO_2_. The cells were split three times a week.

### HCV Grown in Huh-7 Cell Culture (HCVcc)

We used a modified JFH1 (Japanese Fulminant Hepatitis-1) virus containing titer-enhancing mutations (Delgrange et al., 2007) and in which the A4 epitope of HCV glycoprotein E1 of strain H77 was reconstituted (Goueslain et al., 2010). The viral stock was produced in Huh-7 cells. The titer of the stock was 5.10^5^ focus forming unit (ffu)/mL.

### Selection, Collection, and Extraction of Medicinal Plants

Several medicinal plants used in traditional medicine for the healing of liver-related disorders were identified in the Western region of Cameroon. Five of these plants, *Discoglypremna caloneura* (Euphorbiaceae), *T. dregeana* (Meliaceae), *Detarium microcarpum* (Caesalpinaceae), *P. capitata* (Loranthaceae), and *Tapinanthus bangwensis* (Loranthaceae) were investigated for their anti-HCV activity (**Table 1**). These plant materials were collected near Yaounde Central region of Cameroon. After collection, all materials were verified by Mr. Nana, plant taxonomist at the National Herbarium of Cameroon in Yaounde.

**Table 1:** List of the plants used for the preparation of crude extracts tested for their anti-hepatitis C virus (anti-HCV) activity.

Botanical name	Family	Voucher number	Parts	Type of extract	N^o^
*Discoglypremna caloneura*	Euphorbiaceae	4207/SFR/Cam	Stem bark	Crude extract	F1
			Roots	Crude extract	F2
*Trichilia dregeana*	Meliaceae	2126/SFR/Cam	Roots	Crude extract	F3
*Detarium microcarpum*	Caesalpiniaceae	49834/SFR/Cam	Stem bark	Crude extract	F4
*Phragmantera capitata*	Loranthaceae	5068/SFR/Cam	Leaves	Crude extract	F5
*Tapinanthus bangwensis*	Loranthaceae	18628/SFR/Cam	Fruits	Crude extract	F6
			Leaves	Crude extract	F7

The stem and root of *D. caloneura* (Euphorbiaceae) were collected in May 2012 in Ezezang near Yaounde town central region of Cameroon. A voucher specimen was deposited at the National Herbarium of Cameroon under the N° 4207/ SFR/ CAM. Air-dried and powdered stem and root of *D. caloneura* (2,2 and 1,5 kg, respectively) were exhaustively extracted twice at room temperature for 72 h using 98% methanol (static maceration). Each suspension was filtered using Whatman paper 2 and the resulting filtrate was concentrated under vacuum using a rotary evaporator under reduced pressure to give 120 g of brown residue and 70 g of oily material, respectively.

The root of *T. dregeana* (Meliaceae) was collected in July 2011, in Eloumden Mountain near Yaounde town central region of Cameroon. A voucher specimen was deposited at the National Herbarium of Cameroon under the N° 2126/SFR/CAM. Using the same procedure of extraction as above on air-dried and powdered root (3.5 kg), we obtained 200 g of black residue.

The stem bark of *D. microcarpum* (Caesalpiniaceae) was collected in February 2013 in Puma, a locality of the Littoral region of Cameroon. A voucher specimen was deposited at the National Herbarium of Cameroon under the N° 49834/SFR/CAM. Using the same procedure of extraction as above on air-dried and powdered root (4 kg), we obtained 430 g of red residue.

The leaves of *P. capitata* (Loranthaceae) were collected in November 2012 on the Campus of the University of Yaounde I. A voucher specimen was deposited at the National Herbarium of Cameroon under the N° 5068/SFR/CAM. Using the same procedure of extraction as above on air-dried and powdered leaves (2 kg), we obtained 150 g of green residue.

The fruits and leaves of *T. bangwensis* (Loranthaceae) were collected in November 2012 on the Campus of the University of Yaounde I. A voucher specimen was deposited at the National Herbarium of Cameroon under the N° 18628/SFR/CAM. Using the same procedure of extraction as above on air-dried and powdered fruits (1.6 kg) and leaves (1.8 kg), we obtained 500 g of brown residue and 120 g of green residue, respectively.

All the plant extracts were stored at 4°C until their use for biological tests and further phytochemical investigations. The extracts for bioassay were dried in vacuo before being used. A total of seven extracts, F1 to F7 were obtained (**Table 1**).

### HCVcc Inhibition Assay

#### General Procedure

During the inhibition assay, the cells were maintained in an incubator at 37°C with 5% CO_2_. The day before infection, Huh-7 cells were plated in 96-well plates at a concentration of 6.000 cells/well. Huh-7 cells were infected for 2 h with HCVcc at a multiplicity of infection (MOI) of 0.5. The inoculum was removed and cells were overlaid with fresh medium. After 28 h, infected cells were fixed with ice-cold methanol. DMSO (0.01%) was used as a control because this solvent was used to prepare stocks of plant extract.

#### Identification of Active Plant Extracts

For the first screening of the plant extracts, F1, F2, F4, F6, or F7 extract at 25 μg/mL, or F3 or F5 extract at 125 μg/mL, was added to the culture medium during the 2 h inoculation period and the 28 h post-inoculation period.

#### Effect on Virus Entry or Replication Step

To test the effect of the compounds on entry or replication, the same amount of each extract was added either during the 2 h inoculation period, the viral entry step, or the 28 h post-inoculation period, the viral replication step. In parallel, the compounds were added to the culture medium during both inoculation and replication steps, continuously. EGCG, an inhibitor of the entry step (Calland et al., 2012a), was added as a control at 50 μM during the 2 h-inoculation step. Boceprevir, a viral NS3/4A protease inhibitor (Sarrazin et al., 2012), inhibiting the replication step, was added as a control at 1 μM during the 28 h-post-inoculation step.

#### Effect on Virus Assembly/Secretion Step

To test the effect of the compounds on the assembly/secretion step, Huh-7 cells were inoculated for 2 h with HCVcc, the inoculum was removed and replaced with DMEM containing 25 μg/mL of F1, F2, F6, or F7 extract for 46 h. This period of time allows the secretion of viral particles in the medium. The medium containing the virus was collected and used to inoculate Huh-7 cells for 2 h. The inoculum was removed and replaced by culture medium without plant extract for 28 h. Cells were fixed with ice-cold methanol.

### Immunofluorescent Detection Assay

Cells were then processed for immunofluorescent detection of E1 envelope glycoprotein with Cy3-labeled antibody as previously described (Rouille et al., 2006). Nuclei were stained with 1 μg/mL DAPI. Confocal images were recorded on an automated confocal microscope IN Cell Analyzer 6000 (GE Healthcare Life Sciences) using a 20X objective with exposure parameters 405/450 nm and 561/610 nm. Six fields per well were recorded. Each image was then processed using the Columbus image analysis software (Perkin Elmer). Nuclei were first segmented and cytoplasm region extrapolated based on the DAPI staining. Then a cell was considered “infected” for a high ratio of the intensity of the Cy3 staining in the cytoplasm region (relative to that of the nucleus) and Cy3 intensity above a fixed threshold. Quantity of cells per well and MOI were calculated to obtain no more than 40% of infected cells 30 h post infection, allowing the automated quantification. Data were then normalized to DMSO control set-up at 100% of infection.

### Determination of the Half-Maximal Inhibitory Concentrations (IC_50_)

Huh-7 cells were inoculated for 2 h with HCVcc in the presence of 0.1, 1.25, 2.5, 5, 10, 25, 50, 100, 200, 400, or 800 μg/mL of F3 or F5 extract and, 0.01, 0.0625, 0,125, 0.25, 0.5, 1, 2.5, 5, 10, 25, 50, 100 μg/mL of F4 extract. The inoculum was removed and replaced with culture medium without extract for 28 h. Cells were fixed in iced-cold methanol and processed for immunofluorescent detection assay. IC_50_ values were calculated via non-linear regression analysis using a variable-sloe log dose-versus-response curve with a least square (ordinary) using Prism 5 software (GraphPad Software Inc).

### Viability Assay

The day before incubation with the plant extracts, Huh-7 cells were plated in 96-well plates at a density of 6.000 cells/well and placed in the incubator at 37°C with 5% CO_2_. Cells were incubated in 100 μL of culture medium containing different concentrations of the plant extracts (three wells per condition) for either 24, 48, or 72 h in the incubator at 37°C with 5% CO_2_. An MTS [3-(4,5-dimethylthiazol-2-yl)-5-(3-carboxymethoxyphenyl)-2-(4-sulfophenyl)-2H-tetrazolium]-based viability assay (CellTiter 96 AQueous non-radioactive cell proliferation assay, Promega) was conducted as recommended by the manufacturer. Briefly, at the different time points, the cell culture medium was replaced by 100 μl of DMEM and 20 μl of MTS/phenazine methosulfate (PMS) solution, which is transformed in formazan in viable cells, and the plate further incubated at 37°C for 45 min to 2 h. The amount of formazan was quantified by measuring absorbance at 490 nm, recorded using an ELISA plate reader (EL_X_ 808 Bio-Tek Instruments Inc).

### Statistical Analysis

The results were presented as means ± SEM of triplicate experiments. The data were analyzed using Prism statistical software (Graph Pad Inc.) using Mann–Whitney non-parametric test with a confidence interval of 95%. Comparisons were made between each treated group and untreated group (DMSO control). We report two-tailed p-values. Differences between treated and control groups were considered significant for any *p*-value < 0.05. *P*-values were indicated whenever significant differences were observed.

## Results

### Identification of Cameroonian Plant Extracts with Anti-HCV Activity

A first ethnobotanical survey, conducted in the Western region of Cameroon, reported the use of several medicinal plants in traditional medicine for the healing of liver-related disorders. Five of these plants, *D. caloneura*, *T. dregeana*, *D. microcarpum*, *P. capitata*, and *T. bangwensis* were investigated for their anti-HCV activity. Seven crude plant extracts, F1 to F7 were prepared from different parts of these plants (**Table 1**). The HCVcc system used enables analyzing antiviral effect of the compounds during a complete viral life cycle. Plant extracts were solubilized in DMSO at a final concentration of 250 mg/mL and used in anti-HCV experiments at 250 μg/mL in a first set of experiments. This concentration appeared to be toxic for Huh-7 cells for a vast majority of extracts because the total cell number at the end of the experiment was decreased by 99.3, 89.4, 28.3, 61.4, 11.3, 99.3, 98.3%, for F1 to F7 extracts, respectively, compared to DMSO control (data not shown). The concentration was then lowered to 25 μg/mL except for F3 and F5 that were tested at 125 μg/mL because they were less toxic.

**FIGURE 1:**
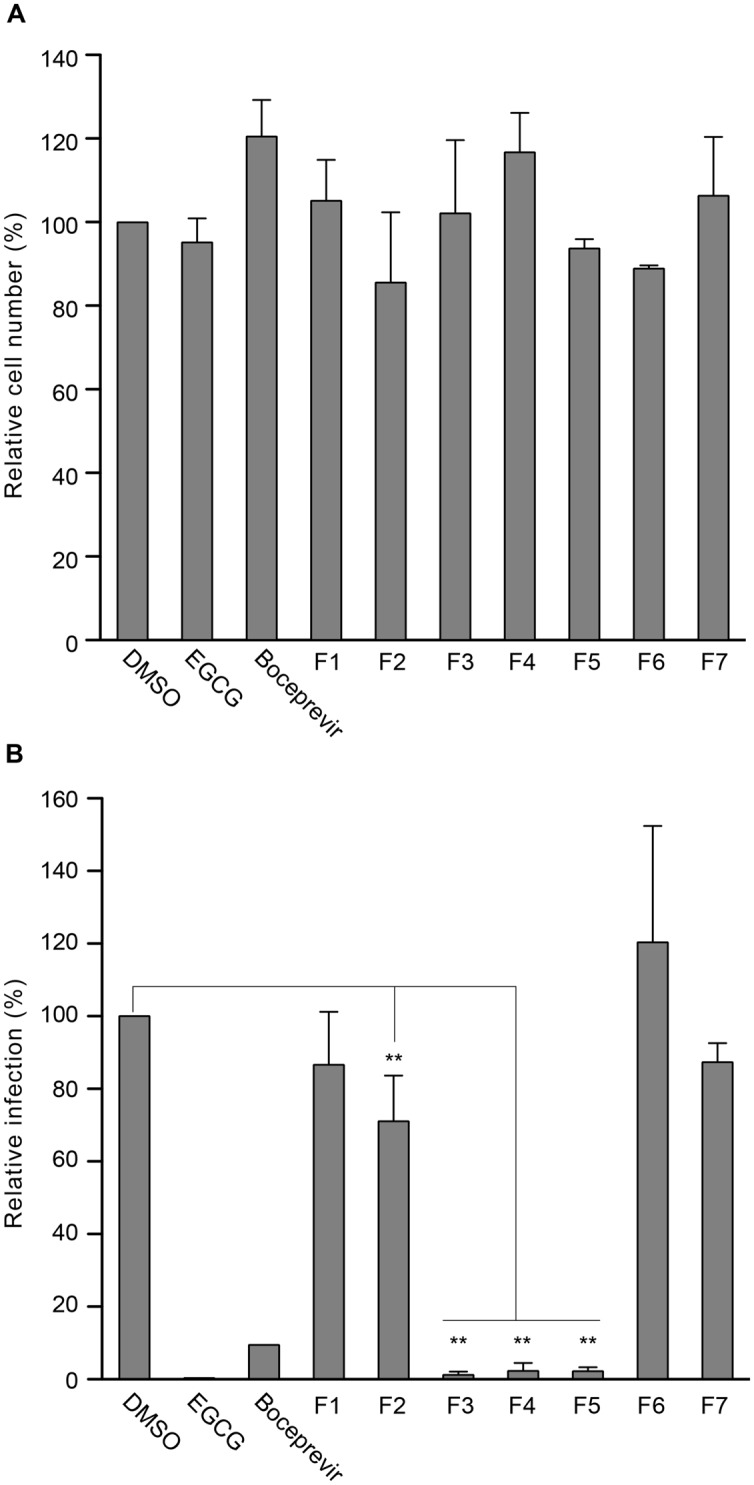
**Screening of plant extracts for anti-hepatitis C virus (anti-HCV) activity.** Huh-7 cells were inoculated with HCV cell culture system (HCVcc) in the presence of plant extracts at 25 or 125 μg/mL for F3 and F5, or 50 μM epigallocatechin gallate (EGCG), or 1 μM boceprevir for 2 h. Dimethyl sulfoxide (DMSO) was used as a control. Cells were further incubated in medium containing plant extracts or molecules for 28 h and cells were fixed. **(A)** Nuclei were stained with 4′,6-diamidino-2-phenylindole (DAPI) to quantify the number of cells. 37,235 cells were quantified in the control. **(B)** Infectivity was measured by the use of immunofluorescence labeling of HCV E1 envelope protein, and by calculating the number of infected cells (33.3 ± 4.4% of infected cells in the DMSO control condition). Data are expressed as a percentage of values measured with DMSO. Data are means of values obtained in three independent experiments performed in triplicate. Error bars represent SEM. Statistical analyses were performed using Mann–Whithney non-parametric test. ^∗∗^*P* < 0.01.

Plant extracts were added during both the 2 h inoculation step and the 28 h post-inoculation step. A 30 h infectious cycle allows us to avoid detecting re-infection events by newly synthesized particles. Infected cells were fixed and submitted to immunofluorescence labeling with an anti-E1 envelope protein antibody (**Figure 1B**). The total number of cells at the end of the experiment was determined by staining of nuclei with DAPI (**Figure 1A**). The results presented in **Figure 1A** show that the compounds were not toxic for the cells at the concentration tested because similar numbers of cells were present in the control and the different plant extract samples. A significant decrease in the number of infected cells was observed for F3, F4 and F5 extracts that showed more than 95% of inhibition in HCV infection at the concentration tested (**Figure 1B**), demonstrating a strong anti-HCV activity for these three plant extracts. A statistically significant decrease in the number of infected cells was also observed for F2 plant extract, (*P* = 0.0022), but with 29% of inhibition of HCV infection, its anti-HCV activity is much less potent than F3, F4, F5 extracts. The three other plant extracts, F1, F6, and F7, displayed infection rates very similar to the control, indicating an absence of significant inhibition of HCV infection. These results indicate that F3 (*T. dregeana* root extract), F4 (*D. microcarpum* stem bark extract), and F5 (*P. capitata* leave extract) contain compounds that strongly inhibit HCV infection.

### Three Plant Extracts Inhibit HCV Entry in Cell Culture

**FIGURE 2:**
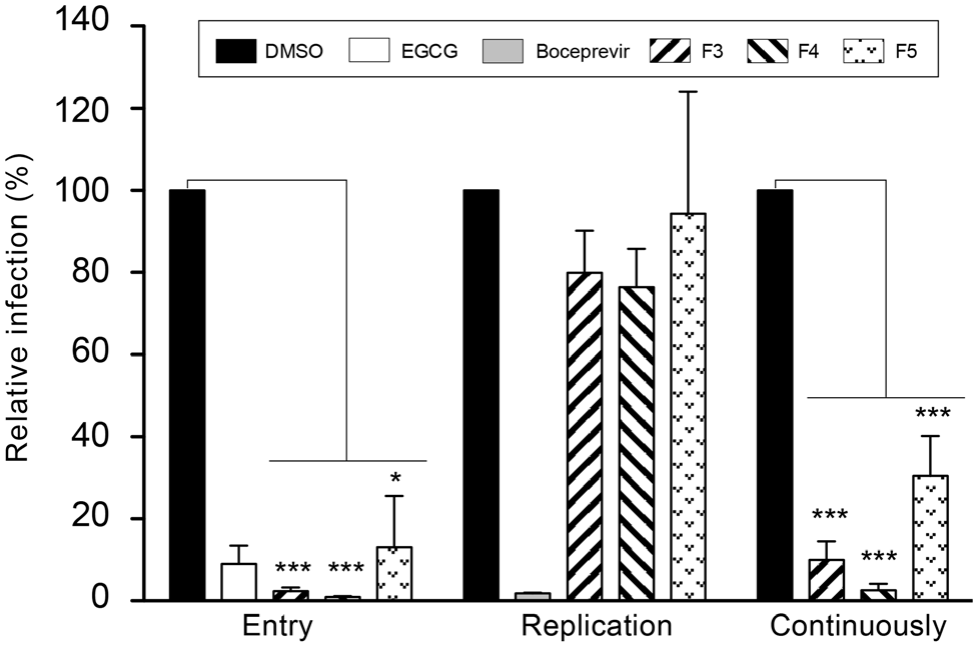
**Time-addition assay of plant extracts during HCV infection.** Huh7 cells were inoculated with HCVcc in the presence (“Entry” and “Continuously”) or absence (“Replication”) of the plant extracts at 125 μg/mL (F3 and F5) or 25 μg/mL (F4). Virus was removed and cells further incubated with culture medium containing the plant extracts (“Replication” and “Continuously”) or without plant extracts (“Entry”). EGCG at 50 μM and boceprevir at 1 μM were added as controls during entry or replication, respectively. Infectivity was measured by the use of immunofluorescence labeling of HCV E1 envelope protein, and by calculating the number of infected cells (24.1 ± 10.2% of infected cells in the DMSO control condition). Data are expressed as a percentage of values measured with DMSO. Data are means of values obtained in three independent experiments performed in triplicate. Error bars represent SEM. Statistical analyses were performed using Mann–Whithney non-parametric test. ^∗^*P* < 0.05, ^∗∗∗^*P* < 0.001.

To further characterize anti-HCV activity of F3, F4, and F5, each extract was added at different steps of the infectious cycle, either during the 2 h-inoculation step which represents the entry step, or during the 28 h post-inoculation step which represents the replication step, or during both steps, continuously. EGCG, an inhibitor of the entry step, and boceprevir, an inhibitor of the replication steps, were added as controls. The results presented in **Figure 2** clearly show a significant decrease in the number of HCV-infected cells when the extracts were added during the inoculation step, as observed with EGCG. Furthermore, no significant antiviral effect was observed when the extracts were added during the replication step. Taken together, these results suggest that F3, F4, and F5 extracts inhibit HCV entry.

### Effect on HCV Particle Release

**FIGURE 3:**
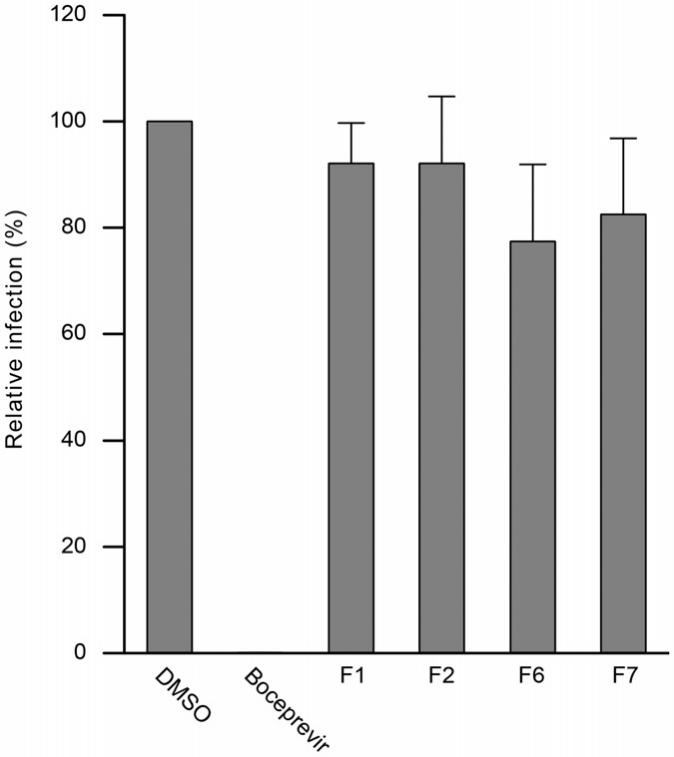
**Effect of plant extracts on HCV secretion.** Huh7 cells were inoculated with HCVcc for 2 h without compounds, virus was removed and plant extracts or fractions were added during 46 h post-inoculation, to allow particle release in the medium. These particles were collected and used to inoculate naive Huh-7 cells for 2 h. Cells were then incubated with culture medium without extracts for 28 h, fixed and subjected to immunofluorescence labeling of E1 envelope protein to quantify infection level. Data are expressed as a percentage of values measured with DMSO (46.7 ± 9.2% of infected cells in the DMSO control condition). Data are means of values obtained in three independent experiments performed in triplicate. Error bars represent SEM.

Extracts that did not exhibit any anti-HCV activity in the first screening, aimed to identify entry or replication inhibitors, could be active in another step of the virus infectious cycle, the assembly/secretion step. To test this hypothesis, plant extracts were added during 46 h post-inoculation, to allow particle release in the medium. These particles were collected and used to inoculate naive Huh-7 cells for 2 h. Cells were then incubated with culture medium without extracts for 28 h, fixed and subjected to immunofluorescence labeling. As shown in **Figure 3**, no significant difference of HCV infection was observed between the control and the different extracts tested showing that none affect HCV particle release. In contrast, when cells were treated post-inoculation with 1 μM boceprevir, a strong inhibition of particle release was observed, as expected, due to the blockade of the replication step.

### Determination of the IC_50_ and the Cytotoxic Effect of the Active Plant Extracts

**FIGURE 4:**
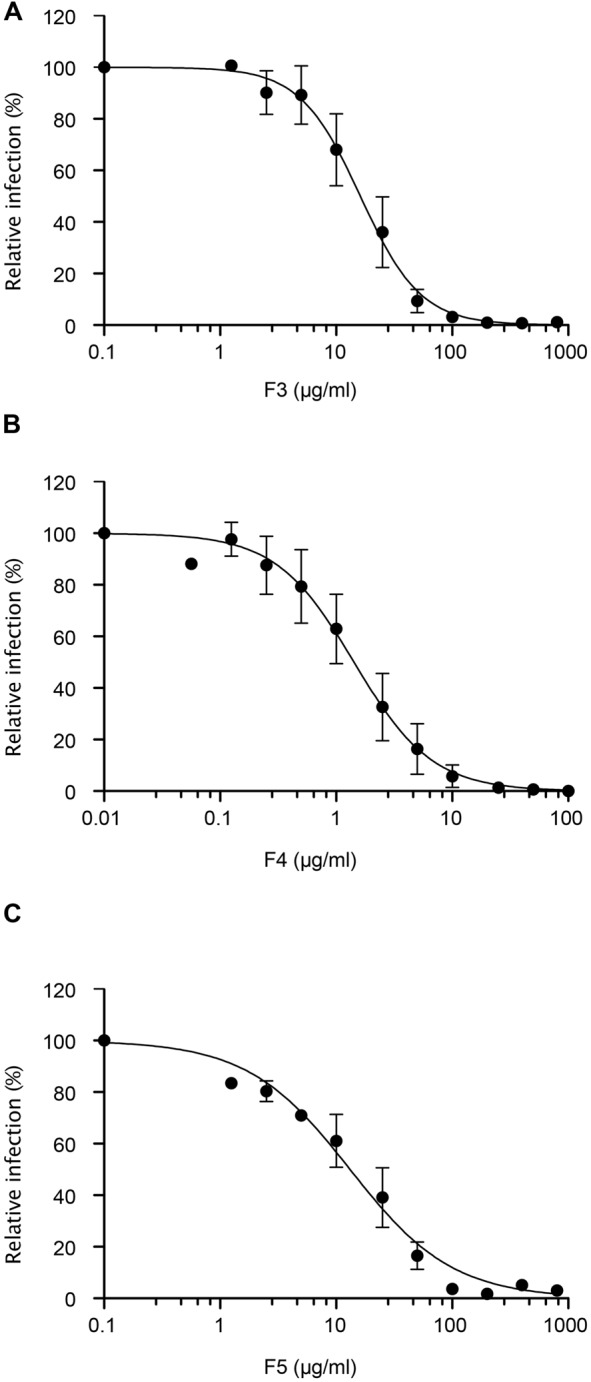
**Dose-response study.** Huh-7 cells were inoculated with HCVcc in the presence of increasing concentration of plant extracts **(A)** F3, **(B)** F4, and **(C)** F5, for 2 h. Inoculum was removed and cells were further incubated with culture medium. Infectivity was measured by the use of immunofluorescence labeling of HCV E1 envelope protein, and by calculating the number of infected cells (14.7 ± 1.6%, 17.9 ± 1.4%, 15,6 ± 1.1% of infected cells in the DMSO control condition for F3, F4, and F5 extracts dose-response assay, respectively). Data are expressed as a percentage of values obtained in the presence of DMSO. Data are means of values obtained in 3 independent experiments performed in triplicate. Error bars represent SEM.

The three plant extracts that were shown to strongly inhibit HCV infection were further analyzed. Their IC_50_ were determined by inoculating Huh-7 cells with HCVcc in the presence of increasing concentrations of each extract (**Figure 4**). All the extracts showed a clear dose-response inhibition of HCV infection confirming their antiviral activity. The IC_50_ values calculated were found to be 16.16, 1.42, and 13.17 μg/mL for F3, F4, and F5, respectively. Taken together these results show that F3, F4, and F5 are inhibitors of HCV infection and that F4 is ten times more active against HCV than F3 and F5.

**FIGURE 5:**
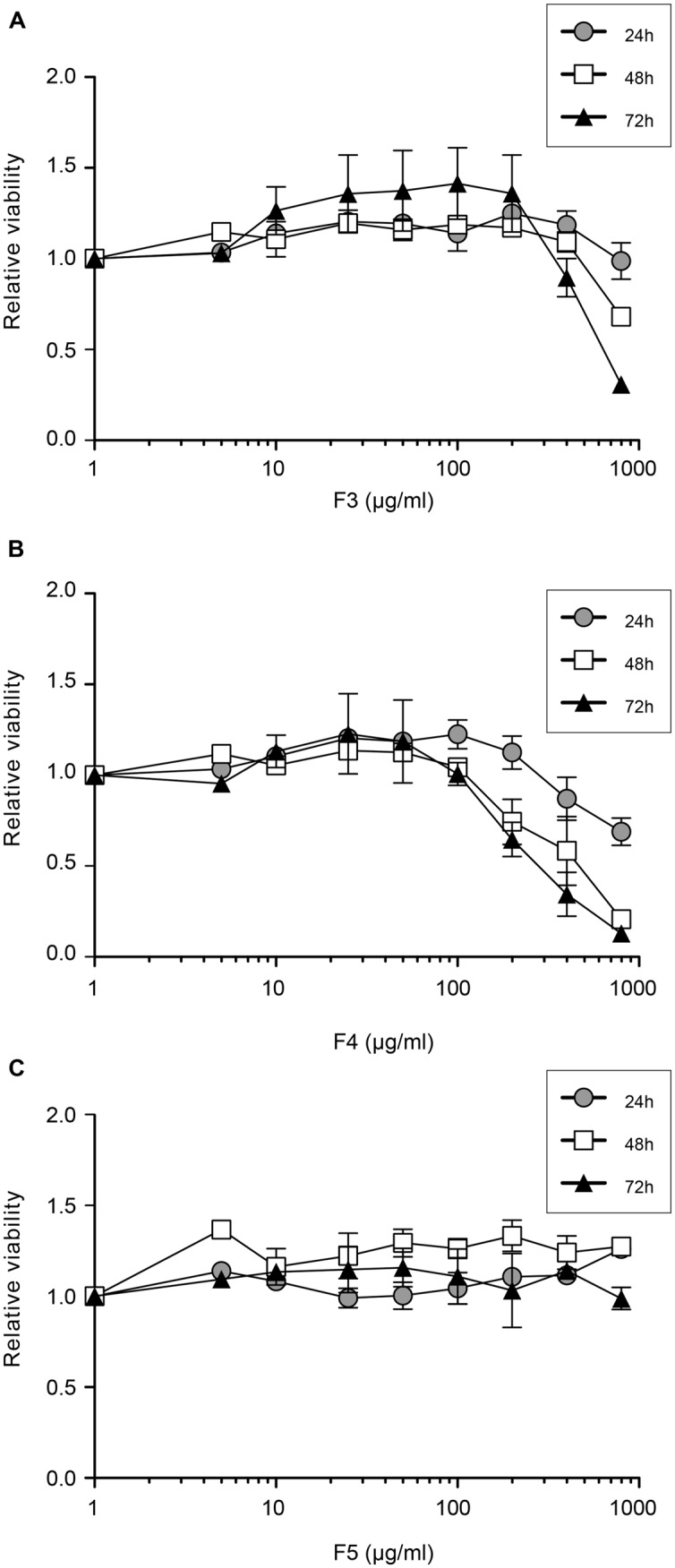
**Cellular toxicity of plant extracts.** Huh-7 cells were cultured in the presence of given concentrations of plant extracts **(A)** F3, **(B)** F4, and **(C)** F5. The viability was monitored using an MTS-based viability assay after 24, 48, and 72 h. Data are expressed as a ratio to control, i.e., the condition without extracts. Data are means of values obtained in three independent experiments performed in triplicate. Error bars represent SEM.

Finally, the cytotoxic effect of F3, F4, and F5 extracts on Huh-7 cells was determined by incubating the cells with increasing concentrations of each plant extract for 24, 48, or 72 h. Cellular viability was determined using a MTS test. The results presented in **Figure 5** show that when incubated with Huh-7 cells for 72 h, F3, F4, and F5, are not toxic for the cells up to 200, 100, and 800 μg/mL (the higher concentration tested), respectively. However, for F3 or F4, the decrease in the relative number of viable cells after 72 h of treatment with 800 μg/mL of extract is not statistically significant. The median-lethal concentration (LC_50_) could not be determined for F5 and was estimated to be 600 μg/mL for F3 and 300 μg/mL for F4. The therapeutic index, which is the LC_50_/IC_50_ ratio, was calculated for F3 and F4 to be approximately 37 and 211 respectively. This index could not be calculated for F5, which was not toxic in Huh-7 cells, but is above 60 (800/13.17).

We estimated the IC_95_ (95% IC) and the IC99 (99% IC) of each extract, allowing 1.5 log and 2 log inhibition of HCV infection in Huh7 cells. For F3, IC_95_, and IC_99_ were estimated to be 100 and 200 μg/mL respectively (**Figure 4A**), concentrations that exhibited no effect on cell viability even for 72 h exposure (**Figure 5A**). For F4, IC_95_, and IC_99_ were estimated to be 15 and 25 μg/mL respectively (**Figure 4B**). These concentrations have no impact on cell viability even for 72 h treatment (**Figure 5B**). For F5, IC_95_, and IC_99_ were estimated to be 250 and 800 μg/mL, respectively (**Figure 4C**). Even if these concentrations are quite high, they have no impact on cell viability (**Figure 5C**).

In conclusion, F3, F4, and F5 are strong inhibitors of HCV infection with IC_50_ between 1 and 16 μg/mL, LC_50_ higher than 300 μg/mL, and therapeutic index between 37 and 211. F4, with an IC_99_ of 25 μg/mL and a therapeutic index of 211, is a very interesting extract.

## Discussion

In this paper, we examined the effect of seven medicinal plants extracts on HCV infection using the HCVcc system. These are plants commonly used in Cameroonian traditional medicine for the treatment of hepatic disorders. Three plants extracts were found very active on this infectious model. These are the methanol extracts from roots of *T. dregeana (Meliaceae),* stem bark of *D. microcarpum (Caesalpiniaceae)* and leaves of *P. capitata* (*Loranthaceae*). These extracts showed a strong inhibition of the viral infection at the different tested concentrations. They have distinguished themselves by their capacity to highly interfere with the HCV life cycle and principally with the entry step. Furthermore, these extracts are not toxic at the active concentration, and display therapeutic indexes between 37 and 211. The four other plant extracts tested were inactive against HCVcc. This could be explained by the differential phytochemical composition and the fact they don’t contain active ingredients found in the F3, F4, and F5 extracts.

The three active extracts identified are HCV entry inhibitors. These inhibitors could be used in combination with other molecules or extracts that inhibit the replication step of the virus like Sofosbuvir or Simeprevir. A multi-drug therapy targeting the different steps of the virus life cycle might overcome the apparition of virus resistant mutants. Furthermore, identification of entry inhibitors is still needed for treating patients undergoing liver transplantation in order to reduce the risk of reinfection of the graft. This reinfection appears systematically after transplantation with adverse outcomes.

Many studies previously reported the antiviral effect of medicinal plants against HCV infection and especially using the HCVcc system. Recently, Wahyuni et al. (2013) investigated the anti-HCV properties of 21 Indonesian medicinal plant ethanol extracts. The authors showed that the presence of some extracts during infection of Huh 7.5 cells with HCVcc genomes of different genotypes resulted in a dose-dependent inhibition of infection. Five plants extracts were identified as the most active ones and some of them like the *Melanolepis multiglandulosa* stem extract and *Ficus fistulosa* leave extract mainly inhibited HCV entry. Our results are similar to these findings but obtained with different plant species.

To best of our knowledge, there are no studies describing the antiviral activities of the three active medicinal plants identified in our study, but information is available on their phyto-chemical composition and related plants. A high polyphenolic content (Lamien-Meda et al., 2008), bioactive diterpenes from fruits (Cavin et al., 2006) and carbohydrates from bark extract (Abreu and Relva, 2002) have been highlighted in *D. microcarpum*. Polyphenols in particular are known to be potent anti-HCV agents, especially flavonoïd compounds (Calland et al., 2012b). Such compounds are not yet isolated in our plant extracts and further experiments are needed to identify the active compound inhibiting HCV entry present in *D. microcarpum*. Previous studies on Loranthaceae showed many antiviral activities. An experimental research on the antiviral potential of ethanol extracts from *Loranthus yadoriki Sieb*, a plant of the Loranthaceae family, demonstrated an inhibitory effect of two components from these extracts on Coxsackie B3 virus (Wang et al., 2000). A recent review on *Loranthus parasiticus Merr*, another Loranthaceae, reported antiviral and antihepatotoxic activities (Moghadamtousi et al., 2014). It was shown, in the study, that the aqueous extract from aerial parts of this plant elicited 59.8% and 27.8% inhibition of HIV-1 protease at doses of 250 and 25 μg/mL, respectively and a significant inhibition of HIV-1-induced cytopathogenicity. Inhibitory activities were also demonstrated on reverse transcriptase of avian myeloblastosis virus with methanol and water extracts. A phytochemical analysis of this plant led to the identification of sesquiterpene lactones such as coriamyrtin, tutin, corianin, and coriatin as the most important group of secondary metabolites. In addition, two known proanthocyanidins and (+)-catechin were identified from the aqueous fraction of *L. parasiticus* leaves by comparing ^1^H NMR and ^13^C NMR spectra with literature values (Wong et al., 2012). *P. capitata* belongs to the same plant family, although differing by the genus. The effects observed with Loranthus species on HIV, Coxsackie and myeloblastosis viruses and here on HCV, confirm the antiviral potential of this plant.

*Trichilia dregeana* is a plant belonging to the Meliaceae family. Plants from this family are commonly used in traditional medicine for the treatment of malaria and inflammation. Anti-HCV properties were also reported with some of them. Wahyuni et al. (2013) showed that the ethanolic extract from *Toona sureni* leaves significantly inhibited the entry and post-entry steps of different HCV strains in cell culture. Wu et al. (2012) identified 3-hydroxy caruilignan C from *Swietenia macrophylla* stems as the most active ingredient of this plant against HCV. The authors showed that this compound inhibited HCV replication and induced IFN responsive genes. The effects observed with *T. dregeana* support these findings and demonstrate the potential of Meliaceae against HCV infection. This result also suggests the presence of flavonolignans- type compounds in this plant although no effect was reported with these compounds on the HCV entry. The antiviral properties of Meliaceae extracts were also found with other viruses. The therapeutic effect of meliacine, an antiviral derived from *Melia azedarach* L., in mice genital herpetic infection were shown for example by Petrera and Coto (2009) and confirmed by Tiwari et al. (2010) who reported the same effect *in vitro* with the neem (*Azadirachta indica* L.) bark extract (NBE) against herpes simplex virus type-1 infection. In that study, Tiwari et al. (2010) showed that NBE significantly blocked HSV-1 entry into cells at concentrations ranging from 50 to 100 μg/mL. Furthermore, authors also found that virions treated with NBE failed to bind the cells, implicating its role as an attachment step blocker. Likewise, cells treated with NBE also inhibited HSV-1 glycoprotein mediated cell-to-cell fusion and polykaryocytes formation, suggesting an additional role of NBE at the viral fusion step.

These results prove the efficacy of plant extracts in inhibiting HCV infection. It would be interesting to identify the active compounds present in these extracts, especially for *D. microcarpum* stem extract that is very active at low concentration (IC_50_ = 1.42 μg/mL, and IC_99_ = 25 μg/mL) and encompasses a therapeutic index of 211. Experiments should be performed to prove the activity of these extracts in pre-clinical models such as primary human hepatocytes or humanized mice. Then, clinical trials could be envisaged to first, demonstrate the innocuousness of the extracts in humans, and second their efficacy against HCV. The studied plants extracts or compounds isolated from these extracts might not be used as unique anti-HCV treatment but in combination with the therapeutic arsenal to reduce the cost of the actual therapy and increase the number of people with access to this therapy.

## Conflict of Interest Statement

The authors declare that the research was conducted in the absence of any commercial or financial relationships that could be construed as a potential conflict of interest.
